# Intra- and Inter-Rater Reliability of a Systematic Video Analysis of ACL Injuries in Elite Men’s Football

**DOI:** 10.3390/sports14070284

**Published:** 2026-07-06

**Authors:** Sara F. Oliveira, Maria M. Castela, Konstantinos Spyrou, António P. Veloso, João Brito

**Affiliations:** 1 Interdisciplinary Center for the Study of Human Performance (CIPER), Faculdade de Motricidade Humana, Universidade de Lisboa, Cruz Quebrada-Dafundo, 1499-002 Lisboa, Portugal; mariamiguelcastella@gmail.com (M.M.C.); apveloso@fmh.ulisboa.pt (A.P.V.); joao.brito@fpf.pt (J.B.); 2 FPF Academy, Portuguese Football Federation, Cruz Quebrada-Dafundo, 1495-433 Oeiras, Portugal; 3 Universidad Católica San Antonio de Murcia Research Center for High Performance Sport, Catholic University of Murcia, 30107 Murcia, Spain; kspyrou@ucam.edu

**Keywords:** anterior cruciate ligament, soccer, observational checklist, situational patterns, injury mechanism

## Abstract

While systematic video analysis is widely used to understand anterior cruciate ligament (ACL) injury mechanisms and contexts, human observation reliability remains a source of concern. This study evaluates the intra- and inter-rater reliability of a systematic video-analysis checklist for assessing ACL injuries in elite men’s football. Twenty-five match-related injuries from the top six European leagues (2020–2024) were randomly selected. Independent observers assessed contextual and situational (sunny weather, match minute, playing phase, field location, injury side, dominant leg, and situational pattern), biomechanical (player contact and anatomical area of player contact), and neurocognitive (attentional inhibition and motor response inhibition) variables. Reliability was calculated using Cohen’s kappa (κ) and Intraclass Correlation Coefficients (ICC). Quantitative variables and macro-contextual factors, including injury side, playing phase, and situational pattern (0.810 < κ < 1.000) revealed near-perfect to perfect agreement. Biomechanical details exhibited substantial agreement (0.601 < κ < 0.784). Neurocognitive variables only reached moderate to substantial agreement (0.503 < κ < 0.752), while visual speed estimations proved highly unreliable (−0.106 < κ < 0.412). The checklist is a highly reliable tool for evaluating the contextual and situational patterns of ACL injuries, but visual speed estimation should be removed or replaced by objective tracking technologies.

## 1. Introduction

Anterior cruciate ligament (ACL) injuries represent one of the most severe and impactful musculoskeletal injuries in professional football [1,2,3,4,5]. These injuries are characterized by long rehabilitation processes and prolonged absences from play, substantial financial burdens for clubs [6], and a significant risk of long-term clinical consequences for athletes, such as subsequent ipsilateral and contralateral injuries [7], early-onset osteoarthritis [8], and decreased career longevity [2,5]. Given the impact of these injuries on professional players, understanding the precise injury mechanisms and the situational patterns surrounding ACL ruptures is a critical first step in developing targeted, evidence-based injury prevention strategies and rehabilitation protocols [9,10].

In recent years, systematic video analysis has emerged as a widely used and effective methodological approach for investigating the mechanisms, movement patterns, player behavior, and biomechanics preceding and during injuries [11]. Several systematic video analyses of ACL injuries across various sports have been published [12,13,14,15], with a number of them focusing on football [7,16,17,18,19,20,21]. By employing structured observational checklists, researchers can categorize situational patterns and broader playing contexts associated with ACL injuries. Previous studies using identical observation checklist methodologies detailed a large number of ACL injuries, injury mechanisms, situational patterns, and kinematics in Italian [7], Spanish [21], and English [16] professional football. While these single-country studies offer important insights, it remains unclear whether their findings regarding injury mechanisms and situational patterns can be generalized across different countries and leagues.

Despite the widespread adoption and clear clinical value of systematic video analysis, the robustness of this methodology inherently relies on the accuracy and consistency of human observers [22,23]. Human judgment is highly variable, and this inconsistency between and within experts can create “noise” and consequently reduce the reliability of evaluative decisions [24]. Observational assessments are vulnerable to subjective interpretation, recall bias, and human error, which can introduce significant variability and compromise the reproducibility of epidemiological data [25,26].

Therefore, the current article aims to evaluate the intra- and inter-rater reliability of a systematic video-analysis checklist for assessing ACL injuries in elite men’s football. By applying this tool to a broad sample of ACL injuries occurring during match play in six European elite male football leagues, this study aimed to examine the consistency of observer assessments across a comprehensive range of epidemiological and contextual variables.

## 2. Materials and Methods

### 2.1. Study Design and Sample Selection

A retrospective observational study was conducted to evaluate the reliability of a previously used systematic video analysis checklist for ACL injuries [7,16]. The variables included in this checklist are based on well-established and previously validated injury mechanisms and situational patterns widely described in the scientific literature [8,27]. A systematic surveillance strategy was adopted using the Noisefeed^®^ platform (Noisefeed V2, Chiavari, Italy), a specialized injury database for professional football, to identify ACL injuries across male professional football players competing in six men’s European leagues (England, Italy, Spain, Germany, France, and Portugal) from 1 August 2020 to 30 April 2024. The database was filtered for “knee” injuries, specifically tagged as “ACL ruptures”, for “match” injuries, and for the seasons under analysis.

Injuries were included if: (1) they involved a complete ACL rupture; (2) they occurred during a match, regardless of the match context, encompassing league and cup matches, European club competitions, international fixtures (national teams), and friendly matches; and (3) broadcast-quality video images of the injury event were available. Exclusion criteria comprised injuries sustained during training sessions or match warm-ups, incomplete or partial injuries managed conservatively, and cases where video footage was not available, was of low resolution, or failed to capture the injury mechanism.

### 2.2. Video Acquisition and Processing

The videos of confirmed injuries were directly analyzed on the Noisefeed platform. Each video was examined from approximately 12–15 s before to 3–5 s after the estimated injury frame (IF), as in previous studies [7,16], to evaluate the playing situation and injury mechanism accurately. The specific biomechanical estimated IF was defined as the point of visible tissue failure or, consistent with previous literature, was estimated at 40 ms following initial contact (IC) of the foot with the ground [7,21]. Video footage relied on standard 2D broadcast data from the main broadcast camera angle. When available, multiple 2D camera angles and slow-motion replays were added to the sequence.

### 2.3. Observational Checklist and Variables

The systematic video analysis observational checklist, available in Table A1 of Appendix A, was designed to capture a comprehensive range of contextual, situational, biomechanical, and anatomical factors. The following variables were collected:

(1) Contextual and situational variables: weather conditions (precipitation and presence of sun); match minute and individual minutes played; playing phase, characterized as defensive or offensive based on ball possession; field location, mapped using a standardized pitch grid dividing the field into defensive, middle, and offensive thirds, intersected by left, central, and right corridors, resulting in six specific zones; injury side based on video data and injury history information; dominant leg, defined as the preferred kicking leg (right or left); player situational pattern (pressing, tackling, being tackled, regaining balance after kicking, landing from jump, or other). However, no statistical analysis could be performed for the precipitation variable due to the total absence of variance in both intra- and inter-rater responses.

(2) Biomechanical and anatomical variables: player contact preceding injury, which was used to identify three different injury mechanisms [(1) non-contact injury, occurring without any contact (at the knee or any other level) before or at IF; (2) indirect contact injury, when the injury results from an external force applied to the player, but not directly to the injured leg; (3) direct contact injury, when an external force directly applied to the injured leg causes the injury]; horizontal and vertical velocities (zero, low, or high) to determine the intensity of the action.

Additionally, two primary aspects of inhibitory control were also analyzed, particularly attentional inhibition, defined as the ability to resist interference from external environmental stimuli by maintaining selective attention on the task-relevant situation [28,29], and motor response inhibition, which refers to the ability to stop unwanted or incorrect motor actions by blocking behaviors and interrupting inappropriate automatic reactions, thereby replacing those responses with better, more thought-out responses adapted to the situation [21,29,30]. For the purpose of video analysis, these neurocognitive concepts were evaluated using observable physical indicators. For example, attentional inhibition was inferred by evaluating whether or not the player’s visual focus and head position changed immediately before the injury. On the other hand, motor response inhibition was inferred by observing evident physical attempts to alter their movement trajectory or to interrupt an action.

### 2.4. Video Analysis Procedures

From the initial cohort of 119 included for analysis, a sub-sample of 25 injury cases was randomly selected to perform the reliability analysis. This represents approximately 21% of the total cohort, providing a highly representative fraction for evaluating observational agreement. This sample size is consistent with established methodological guidelines for reliability studies involving two raters and expecting high reliability coefficients, which suggest that 20 to 30 heterogeneous cases are sufficient to achieve adequate statistical power when high reliability coefficients are expected [25,31]. However, because the checklist includes several categorical variables with multiple response options, we acknowledge that kappa estimates can be sensitive to low or unbalanced category frequencies. Therefore, the exact distribution of intra- and inter-rater responses across all categories is reported in Appendix B Table A2 and Table A3.

The randomization procedure was performed using the Google generative artificial intelligence assistant, Google Gemini 3.1 Pro. Specifically, the complete list of unique injury identification numbers (IDs) corresponding to the initial cohort was provided to the AI model, along with a prompt instruction to randomly select 25 distinct IDs. Concerning neurocognitive variables, another subsample of 25 injuries was randomly selected using the same method, but this time from among the 89 non-contact and indirect contact injuries, as the 25 previously selected for the remaining variables included 125 direct contact ACL injuries, for which no neurocognitive variable assessment was performed.

Two independent observers (SO and KS for attentional inhibition and motor response inhibition, and SO and MC for the remaining variables) evaluated the video footage according to a predetermined checklist (Table A1). The evaluation team members have diverse professional backgrounds, including biomedical engineering (SO), sports science (KS), and epidemiology, biostatistics, and research in health (MC, currently a medical student). Before data collection, the raters underwent a familiarization process at a dedicated consensus meeting to review the checklist, align operational definitions, and establish standardized evaluation criteria. During the formal evaluations, all raters had access to the exact same video clips, camera angles, and slow-motion replay conditions on the platform to ensure standardized observational conditions. The observers were blinded to each other’s assessments to prevent bias. To assess intra-rater reliability, a repeated assessment occurred after at least a 30-day interval to minimize recall bias, consistent with standard methodological recommendations regarding memory effects in observational reliability studies [32].

### 2.5. Ethical Considerations

This study utilized publicly available video footage from televised professional football matches and data from a commercial subscription platform accessible for analysis purposes. No direct interaction with human participants occurred, no clinical interventions were performed, and no private health records were accessed. All player data were strictly anonymized during the analysis phase. Accordingly, since the research involved only the secondary analysis of publicly broadcast video records, ethical approval and informed consent were not required. However, the ethical principles of the Declaration of Helsinki were considered where relevant to the conduct of the research. Moreover, general ethical principles regarding research integrity, data protection, and confidentiality were strictly respected throughout the study.

### 2.6. Statistical Analysis

Statistical analyses were performed using JASP statistics software (v0.97.4, JASP Team, 2025). Descriptive statistics were used to characterize the sample.

For categorical variables, intra- and inter-rater agreements were calculated using Cohen’s kappa coefficient, with corresponding 95% Confidence Intervals (95% CI). Cohen’s kappa values were interpreted as follows: no (0), slight (0.01–0.20), fair (0.21–0.40), moderate (0.41–0.60), substantial (0.61–0.80), near-perfect (0.81–0.99), and perfect agreement (1.00) [33].

For quantitative variables, data distribution was first assessed for normality using the Shapiro–Wilk test, and variance homogeneity was confirmed using Levene’s test. Subsequently, reliability was determined using Intraclass Correlation Coefficient (ICC), specifically two-way mixed models with absolute agreement (ICC 3,1), reported alongside their 95% CIs. ICCs were interpreted as follows: poor (<0.50), moderate (0.50–0.75), good (0.75–0.90), and excellent (>0.90) [25,26].

## 3. Results

Table 1 and Table 2 present the reliability values (Cohen’s kappa and ICC), their respective 95% CIs, and interpretations for all assessed variables.

### 3.1. Intra-Rater Reliability

Repeated assessments by the same observer yielded near-perfect to perfect agreement for most variables. For quantitative variables, preliminary assessment confirmed that the data met the assumptions of normality ($p_{\mathrm{match}\;\min\;\mathrm{obs}.\;1}$ = 0.961; $p_{\mathrm{match}\;\min\;\mathrm{obs}.\;2}$ = 0.961; $p_{\mathrm{individual}\;\min\;\mathrm{obs}.\;1}$ = 0.950; $p_{\mathrm{individual}\;\min\;\mathrm{obs}.\;2}$ = 0.951) and homogeneity ($p_{\mathrm{match}\;\min}$ = 0.999; $p_{\mathrm{individual}\;\min}$ = 0.997). The ICC showed absolute excellent reliability for both the match minute (ICC = 1.000, 95% CI: [1.000–1.000]) and the individual minutes played (ICC = 1.000, 95% CI: [1.000–1.000]).

Regarding categorical variables, Cohen’s kappa analysis demonstrated perfect agreement (κ = 1.000, 95% CI: [1.000, 1.000]) for injury side, dominant leg, playing phase, and both field location third and corridor. As previously stated, reliability for precipitation could not be estimated due to a total lack of variability. Near-perfect agreement was achieved for player initial contact (κ = 0.915, 95% CI: [0.753, 1.000]), injury classification (κ = 0.879, 95% CI: [0.718, 1.000]), situational pattern (κ = 0.898, 95% CI: [0.763, 1.000]), and feet on the ground (κ = 0.834, 95% CI: [0.617, 1.000]). Biomechanical details, specifically anatomical area of the initial contact (κ = 0.773, 95% CI: [0.487, 1.000]), anatomical area at the injury frame (κ = 0.601, 95% CI: [0.357, 0.845]), and leg loading (κ = 0.784, 95% CI: [0.378, 1.000]), showed substantial agreement, as well as the presence of sun (κ = 0.606, 95% CI: [0.305, 0.906]). Neurocognitive variables, namely attentional (κ = 0.590, 95% CI: [0.271, 0.910]) and motor response inhibition (κ = 0.503, 95% CI: [0.019, 0.988]), only reached moderate agreement. Conversely, visual speed estimation proved highly problematic. Vertical speed demonstrated only fair agreement (κ = 0.346, 95% CI: [0.034, 0.657]), while horizontal speed estimation yielded a negative coefficient (κ = −0.106, 95% CI: [−0.207, −0.005]), indicating a worse-than-chance level of disagreement.

### 3.2. Inter-Rater Reliability

When comparing evaluations from two independent observers, the checklist maintained a high level of reliability. Quantitative assessment showed excellent reliability for the match minute (ICC = 1.000, 95% CI: [1.000, 1.000]) and good reliability for individual minutes played (ICC = 0.885, 95% CI: [0.760, 0.947]), again after confirming the assumption of normality ($p_{\mathrm{match}\;\min\;\mathrm{rater}\;1}$ = 0.958; $p_{\mathrm{match}\;\min\;\mathrm{rater}\;2}$ = 0.958; $p_{\mathrm{individual}\;\min\;\mathrm{rater}\;1}$ = 0.955; $p_{\mathrm{individual}\;\min\;\mathrm{rater}\;2}$ = 0.961) and homogeneity ($p_{\mathrm{match}\;\min}$ = 0.993; $p_{\mathrm{individual}\;\min}$ = 0.981).

Categorical agreement remained perfect (κ = 1.000, 95% CI: [1.000, 1.000]) for injury side, dominant leg, and playing phase, but both the field location third (κ = 0.810, 95% CI: [0.609, 1.000]) and corridor (κ = 0.816, 95% CI: [0.623, 1.000]) only reached near-perfect agreement. Similarly, the inter-rater reliability for precipitation could not be estimated due to a lack of variance. Near-perfect inter-rater agreement was also achieved for other variables: player initial contact (κ = 0.918, 95% CI: [0.761, 1.000]), injury classification (κ = 0.878, 95% CI: [0.718, 1.000]), situational pattern (κ = 0.948, 95% CI: [0.849, 1.000]), and leg loading (κ = 0.841, 95% CI: [0.538, 1.000]). The anatomical area of initial contact (κ = 0.682, 95% CI: [0.395, 0.969]), the anatomical area of the indirect contact at the injury frame (κ = 0.759, 95% CI: [0.562, 0.958]), and the number of feet on the ground (κ = 0.613, 95% CI: [0.562, 0.958]) revealed substantial agreement. Neurocognitive variables only reached substantial agreement for motor response inhibition (κ = 0.752, 95% CI: [0.434, 1.000]) and moderate agreement for attentional inhibition (κ = 0.590, 95% CI: [0.163, 0.271]). The evaluation of the presence of the sun dropped to moderate agreement (κ = 0.401, 95% CI: [0.090, 0.713]). Similar to the intra-rater results, visual speed estimation was unreliable between different observers, with horizontal speed showing moderate agreement (κ = 0.412, 95% CI: [−0.053, 0.876]) and vertical speed demonstrating only fair agreement (κ = 0.380, 95% CI: [0.108, 0.651]).

## 4. Discussion

This study aimed to evaluate the intra- and inter-rater reliability of a systematic video analysis checklist designed to assess the mechanisms, situational patterns, and contextual factors of ACL injuries in elite male professional football. Systematic video-analysis methodologies and observational reliability assessments have been widely and successfully explored within the sports medicine and biomechanics literature. However, to the best of our knowledge, this is the first study to assess the intra- and inter-rater reliability of this specific comprehensive observational checklist for assessing ACL injury mechanisms, situational patterns, and contextual factors through video analysis in elite men’s football. Overall, the main findings demonstrate that the systematic checklist is a highly reliable tool for most variables, revealing substantial to perfect agreement, although neurocognitive factors proved more challenging to assess consistently. However, the study also highlights a critical methodological limitation in observational video analysis: the visual estimation of player speed is highly unreliable and should be interpreted with extreme caution in future systematic video-analysis studies.

The results demonstrated remarkable consistency for exact quantitative values (ICC = 0.885 to 1.000) and macro-contextual categorical values, including injury side, dominant leg, playing phase, field location, player IC, injury classification, and situational pattern (κ = 0.810 to 1.000) in both intra- and inter-rater assessments. Methodological literature supports the idea that variables assessed on nominal scales with discrete categories are more easily standardized, thus generating near-perfect levels of agreement [25,26,32]. This validates the use of this checklist to accurately characterize the generic mechanism and tactical situation of ACL injuries in elite football, aligning with previous observational studies that categorize situational patterns [7,16,21]. The reliability coefficients reported for this system are comparable to, or higher than, those reported for other 2D video-based scoring systems in sports medicine. For instance, the Cutting Movement Assessment Score (CMAS) demonstrated excellent intra-rater reliability (ICC = 0.96) and moderate inter-rater reliability (ICC = 0.67) [34]. Similarly, the Quality Appraisal for Sports Injury Video Analysis Studies (QA-SIVAS) scale recently exhibited excellent inter- and intra-rater reliability (ICC > 0.97) [35]. Consequently, the checklist meets a high standard in the domain of observational video analysis.

Establishing this high reliability is not merely a methodological exercise, it has significant clinical and practical implications. Consistent and precise video analysis allows sports medicine professionals to accurately identify risky movement patterns and contextual triggers that precede ACL injuries. These robust data can then be translated into practical feedback for coaches and athletes, who can implement targeted technique corrections during training, adjust tactical behaviors that expose players to vulnerable positions, and ultimately improve the early detection of other risk situations, creating a more effective feedback loop for injury mitigation strategies.

Despite high reliability in classifying the general injury mechanism, variables focusing on specific biomechanical details, such as anatomical area of initial contact ($\kappa_{intra\text{-}rater}$ = 0.773 and $\kappa_{inter\text{-}rater}$ = 0.682), anatomical area at the injury frame ($\kappa_{intra\text{-}rater}$ = 0.601 and $\kappa_{inter\text{-}rater}$ = 0.759), leg loading ($\kappa_{intra\text{-}rater}$ = 0.784 and $\kappa_{inter\text{-}rater}$ = 0.841), and feet on the ground ($\kappa_{intra\text{-}rater}$ = 0.834 and $\kappa_{inter\text{-}rater}$ = 0.613), showed slightly lower agreement, falling mostly into the “substantial agreement” category. Previous literature evaluating observational biomechanical methods corroborates that visual assessment of specific postures or joint kinematics is inherently more difficult and susceptible to human error [22,23]. In the real-game context, this challenge is aggravated by the high movement speeds, player occlusion, and suboptimal broadcast camera angles. Nevertheless, the substantial agreement obtained demonstrates that it is still possible to extract reliable postural data from television broadcasts for most injuries.

The assessment of neurocognitive variables presented an additional layer of complexity in observational analysis. Intra-rater agreement only reached moderate agreement, while inter-rater agreement was slightly better, reaching substantial agreement for motor response inhibition but remaining moderate for attentional inhibition. These findings suggest that evaluating internal cognitive processes, such as player focus or decision-making to inhibit a motor response, based on 2D video footage, is inherently subjective. Despite the consensus meeting and familiarization with the checklist, the evaluation of internal cognitive processes based solely on 2D broadcast video footage remains inherently subjective, as raters are required to infer internal neurocognitive processes from contextual behavioral cues rather than from directly measurable features. This discrepancy is likely to account for the lower consistency observed for these variables compared to macro-contextual ones. To improve the robustness of these parameters, future studies could explore indirect quantitative indicators, such as calculating the time elapsed until an action occurs, based on the video’s frame rate, which could serve as a more objective measure of attentional inhibition. Additionally, the potential of more detailed decision trees or enhanced rater training might be explored to further enhance agreement for these concepts.

The most notable finding of this reliability analysis was the poor performance of visual speed estimation. Both horizontal and vertical speed estimations yielded fair to moderate agreement between different raters, and even worse agreement when evaluated by the same rater after a 30-day interval. From a statistical perspective, a negative Kappa value indicates an agreement that is worse than what would be expected purely by chance [33,36]. This rarely occurs in clinical contexts, but it indicates that the assessments were completely inconsistent and lacked any systematic pattern of agreement between observations. This result categorically demonstrates that the human eye is unable to reliably quantify or categorize dynamic speed from two-dimensional broadcast images. Previous research has already highlighted the risks associated with visual estimation of complex kinematics from standard video without multidimensional calibration [37]. Consequently, it is strongly recommended that visual speed estimation be removed or substantially modified in future versions of systematic video analysis checklists, as it cannot be treated with the same level of confidence as contextual variables. Researchers should replace subjective visual assessment with objective data driven from synchronized optical tracking technologies, GPS wearable microtechnology, or specialized computer vision algorithms. Alternatively, in the absence of objective tracking data, future observational methodologies could explore deducing acceleration or deceleration indirectly by evaluating qualitative changes in body posture across consecutive frames. However, whether combining these pieces of observational information improves intra- and inter-rater agreement remains a hypothesis and requires formal validation.

Our research supports the validity of using standardized observational checklists to decode ACL injury mechanisms. However, to maximize data quality in future injury surveillance systems, a pre-analysis training period should be implemented. Furthermore, checklists must be refined to exclude or modify variables that are highly dependent on subjective spatiotemporal estimation, such as speed, focusing instead on variables that describe observable body configurations and situational contexts. Though, it should be noted that the current study focused exclusively on male professional football players competing in six European leagues, which may limit the generalizability and applicability of the results, as they may differ in lower divisions or women’s football. Moreover, injury situations could only be analyzed from a single camera view, sometimes with limited image quality, especially when far from the midline camera position. Finally, although the random selection of injury cases was conducted using an AI model to avoid human selection bias, the lack of a fixed mathematical seed in the conversational interface means the exact algorithmic replication of the specific draw cannot be guaranteed, representing a minor methodological limitation regarding perfect reproducibility.

## 5. Conclusions

The systematic video analysis checklist for ACL injuries is a highly reliable methodological tool for evaluating contextual factors, injury mechanisms, and situational patterns in professional football. The findings demonstrate a high degree of consistency, both over time and between independent raters. Nevertheless, neurocognitive variables proved more complex to assess using only standard 2D broadcast video footage, and the visual estimation of player speed appeared highly unreliable and should be replaced by objective measurement technologies. The validation of these observational tools is a crucial step in ensuring high-quality, precise, and reproducible systematic video analysis data, which can robustly support future epidemiological and biomechanical research in sports medicine and injury prevention.

## Figures and Tables

**Table 1:** Intra-rater reliability values (Cohen’s kappa and ICC) for the assessed variables, their 95% CIs, and interpretation.

Variable	Cohen’s Kappa (κ)	95% CI	Interpretation
Presence of the sun	0.606	[0.305, 0.906]	substantial agreement
Injury side	1.000	[1.000, 1.000]	perfect agreement
Dominant leg	1.000	[1.000, 1.000]	perfect agreement
Playing phase	1.000	[1.000, 1.000]	perfect agreement
Field location third	1.000	[1.000, 1.000]	perfect agreement
Field location corridor	1.000	[1.000, 1.000]	perfect agreement
Player IC	0.915	[0.753, 1.000]	near-perfect agreement
Where (player IC)?	0.773	[0.487, 1.000]	substantial agreement
Injury classification	0.879	[0.718, 1.000]	near-perfect agreement
Where (indirect contact at injury frame)?	0.601	[0.357, 0.845]	substantial agreement
Situational pattern	0.898	[0.763, 1.000]	near-perfect agreement
Leg loading	0.784	[0.378, 1.000]	substantial agreement
Feet on the ground	0.834	[0.617, 1.000]	near-perfect agreement
Attentional inhibition	0.590	[0.271, 0.910]	moderate agreement
Motor response inhibition	0.503	[0.019, 0.988]	moderate agreement
Horizontal speed	−0.106	[−0.207, −0.005]	no agreement
Vertical speed	0.346	[0.034, 0.657]	fair agreement
	**ICC**	**95% CI**	**Interpretation**
Match minute	1.000	[1.000, 1.000]	excellent
Individual minutes played	1.000	[1.000, 1.000]	excellent

*Abbreviations:* IC, initial contact; ICC, intraclass correlation coefficient; CI, confidence interval.

**Table 2:** Inter-rater reliability values (Cohen’s kappa and ICC) for the assessed variables, their 95% CIs, and interpretation.

Variable	Cohen’s Kappa (κ)	95% CI	Interpretation
Presence of the sun	0.401	[0.090, 0.713]	moderate agreement
Injury side	1.000	[1.000, 1.000]	perfect agreement
Dominant leg	1.000	[1.000, 1.000]	perfect agreement
Playing phase	1.000	[1.000, 1.000]	perfect agreement
Field location third	0.810	[0.609, 1.000]	near-perfect agreement
Field location corridor	0.816	[0.623, 1.000]	near-perfect agreement
Player IC	0.918	[0.761, 1.000]	near-perfect agreement
Where (player IC)?	0.682	[0.395, 0.969]	substantial agreement
Injury classification	0.878	[0.718, 1.000]	near-perfect agreement
Where (indirect contact at injury frame)?	0.759	[0.562, 0.958]	substantial agreement
Situational pattern	0.948	[0.849, 1.000]	near-perfect agreement
Leg loading	0.841	[0.538, 1.000]	near-perfect agreement
Feet on the ground	0.613	[0.333, 0.893]	substantial agreement
Attentional inhibition	0.590	[0.163, 0.271]	moderate agreement
Motor response inhibition	0.752	[0.434, 1.000]	substantial agreement
Horizontal speed	0.412	[−0.053, 0.876]	moderate agreement
Vertical speed	0.380	[0.108, 0.651]	fair agreement
	**ICC**	**95% CI**	**Interpretation**
Match minute	1.000	[1.000, 1.000]	excellent
Individual minutes played	0.885	[0.760, 0.947]	good

*Abbreviations:* IC, initial contact; ICC, intraclass correlation coefficient; CI, confidence interval.

## Data Availability

Raw videos and data supporting the findings of this study are publicly available on the platform. The systematic analysis data are available in a publicly accessible repository (https://osf.io/56nb3/overview?view_only=a557fc68c1434ce4be249e4b6fc3a8ea, accessed on 26 May 2026).

## Abbreviations

The following abbreviations are used in this manuscript:

ACL	Anterior Cruciate Ligament
ID	Identification Number
IF	Injury Frame
IC	Initial Contact
ICC	Intraclass Correlation Coefficients

## Appendix A

**Table A1:** Observational checklist for systematic video analysis.

Variables	Categories
Weather Conditions	
Precipitation	Yes/No/Unsure
Presence of sunny weather	Yes/No/Unsure
Injury side	Right/Left
Dominant leg injured	Yes/No
Minute zone during the match	0–5/5–10/10–15/15–20/20–25/25–30/30–35/35–40/40–45/45–50/50–55/55–60/60–65/65–70/70–75/75–80/80–85/85–90/90+
Minutes of effective gameplay	0–5/5–10/10–15/15–20/20–25/25–30/30–35/35–40/40–45/45–50/50–55/55–60/60–65/65–70/70–75/75–80/80–85/85–90/90+
Playing phase before injury	Defensive/Offensive
Field location at injury	Defensive third/Midfield third/Offensive third Left side corridor/Middle corridor/Right side corridor
Player situational pattern (only for indirect and non-contact injuries)	Pressing/tackling/being tackled/landing from a jump/regaining balance after kicking/other
Player contact preceding injury	Yes/No
If contact, where?	Upper body/Pelvis/Injured leg/Uninjured leg
Injury classification	Direct contact/Indirect contact/Non-contact
If indirect contact at IF, where?	Upper body/Pelvis/Injured leg/Uninjured leg
Leg loading at IF	Injured leg/Uninjured leg/Unsure
Feet on the ground	One/Two/Unsure
Neurocognitive analysis	
Attentional inhibition	Yes/No
Motor response inhibition	Yes/No
Speed	
Horizontal speed	Zero/Low/High
Vertical speed	Zero/Low/High

*Abbreviations:* IF, injury frame.

## Appendix B

**Table A2:** Distribution of categorical responses across timepoint assessments for intra-rater reliability subsample (n = 25).

Variables	Category	Observation 1	Observation 2
Presence of sunny weather	Yes	14	11
	No	11	14
	Unsure	0	0
Injury side	Right	12	12
	Left	13	13
Dominant leg injured	Yes	15	15
	No	10	10
Playing phase before injury	Defensive	12	12
	Offensive	13	13
Field location third	Defensive third	5	5
	Midfield third	6	6
	Offensive third	14	14
Field location corridor	Left side corridor	7	7
	Middle corridor	10	10
	Right side corridor	8	8
Player situational pattern	Pressing	4	5
	Tackling	4	5
	Being tackled	7	7
	Landing from a jump	3	3
	Regaining balance after kicking	0	0
	Other	7	6
Player contact preceding injury	Yes	15	16
	No	10	9
If contact, where?	Upper body	7	8
	Pelvis	0	0
	Injured leg	6	7
	Uninjured leg	2	1
Injury classification	Direct contact	6	7
	Indirect contact	9	9
	Non-contact	10	9
If contact at IF, where?	Upper body	4	1
	Pelvis	0	0
	Injured leg	6	8
	Uninjured leg	2	1
Leg loading at IF	Injured leg	22	23
	Uninjured leg	1	1
	Unsure	2	1
Feet on the ground	One	14	16
	Two	11	9
	Unsure	0	0
Attentional inhibition	Yes	10	11
	No	15	14
Motor response inhibition	Yes	3	4
	No	22	21
Horizontal speed	Zero	0	0
	Low	3	2
	High	22	23
Vertical speed	Zero	7	2
	Low	14	18
	High	4	5

*Abbreviations:* IF, initial contact.

**Table A3:** Distribution of categorical responses across timepoint assessments for inter-rater reliability subsample (n = 25).

Variables	Category	Rater 1	Rater 2
Presence of sunny weather	Yes	14	9
	No	11	15
	Unsure	0	1
Injury side	Right	12	12
	Left	13	13
Dominant leg injured	Yes	15	15
	No	10	10
Playing phase before injury	Defensive	12	12
	Offensive	13	13
Field location third	Defensive third	5	7
	Midfield third	6	7
	Offensive third	14	11
Field location corridor	Left side corridor	7	6
	Middle corridor	10	12
	Right side corridor	8	7
Player situational pattern	Pressing	4	4
	Tackling	4	4
	Being tackled	7	7
	Landing from a jump	3	2
	Regaining balance after kicking	0	0
	Other	7	8
Player contact preceding injury	Yes	15	14
	No	10	11
If contact, where?	Upper body	7	3
	Pelvis	0	2
	Injured leg	6	7
	Uninjured leg	2	2
Injury classification	Direct contact	6	7
	Indirect contact	9	7
	Non-contact	10	11
If contact at IF, where?	Upper body	4	2
	Pelvis	0	2
	Injured leg	6	7
	Uninjured leg	2	2
Leg loading at IF	Injured leg	22	21
	Uninjured leg	1	2
	Unsure	2	2
Feet on the ground	One	14	9
	Two	11	16
	Unsure	0	0
Attentional inhibition	Yes	10	11
	No	15	14
Motor response inhibition	Yes	4	6
	No	21	19
Horizontal speed	Zero	0	0
	Low	3	5
	High	22	20
Vertical speed	Zero	7	14
	Low	14	8
	High	4	3

*Abbreviations:* IF, initial contact.
